# Role of DUOX in gut inflammation: lessons from *Drosophila* model of gut-microbiota interactions

**DOI:** 10.3389/fcimb.2013.00116

**Published:** 2014-01-10

**Authors:** Sung-Hee Kim, Won-Jae Lee

**Affiliations:** ^1^School of Biological Science and Institute of Molecular Biology and Genetics, Seoul National UniversitySeoul, South Korea; ^2^National Creative Research Initiative Center for Symbiosystem, Seoul National UniversitySeoul, South Korea

**Keywords:** dual oxidase, gut immunity, epithelial cell renewal, gut microbiota, uracil, reactive oxygen species, gut-microbe interactions

## Abstract

It is well-known that certain bacterial species can colonize the gut epithelium and induce inflammation in the mucosa, whereas other species are either benign or beneficial to the host. Deregulation of the gut-microbe interactions may lead to a pathogenic condition in the host, such as chronic inflammation, tissue injuries, and even cancer. However, our current understanding of the molecular mechanisms that underlie gut-microbe homeostasis and pathogenesis remains limited. Recent studies have used *Drosophila* as a genetic model to provide novel insights into the causes and consequences of bacterial-induced colitis in the intestinal mucosa. The present review discusses the interactions that occur between gut-associated bacteria and host gut immunity, particularly the bacterial-induced intestinal dual oxidase (DUOX) system. Several lines of evidence showed that the bacterial-modulated DUOX system is involved in microbial clearance, intestinal epithelial cell renewal (ECR), redox-dependent modulation of signaling pathways, cross-linking of biomolecules, and discrimination between symbionts and pathogens. Further genetic studies on the *Drosophila* DUOX system and on gut-associated bacteria with a distinct ability to activate DUOX may provide critical information related to the homeostatic inflammation as well as etiology of chronic inflammatory diseases, which will enhance our understanding on the mucosal inflammatory diseases frequently observed in the microbe-contacting epithelia of humans.

## Introduction

Bacteria heavily colonize multiple sites in our body. These sites include various mucosal epithelia such as the respiratory, gastrointestinal, and urogenital tracts. It is now evident that commensal community members form an ecosystem in these sites and that this microbial ecosystem impacts diverse ranges of the host physiology (Turnbaugh et al., 2006; Ryu et al., 2008; Garrett et al., 2010; Shin et al., 2011; Storelli et al., 2011). In particular, in the intestine of human beings, approximately one hundred trillion bacterial cells can be found (Gill et al., 2006; Qin et al., 2010). Because any eukaryotic organ readily responds to bacteria by mounting acute inflammation, one of the most important questions is how host mucosal epithelia that are in continuous contact with a diverse range of bacteria manage such microbial burdens. Recent studies in different animal models demonstrated the reciprocal interactions between gut microbiota and the host innate immunity, where the host immunity controls the community of gut-contacting bacteria that in turn modulates the host immunity (Artis, 2008; Ryu et al., 2008; Round and Mazmanian, 2009; Cerf-Bensussan and Gaboriau-Routhiau, 2010; Littman and Pamer, 2011; Maslowski and Mackay, 2011; Hooper et al., 2012). The balanced interactions between the host immunity and the gut-associated bacteria are of central importance to achieve host-microbe symbiosis. However, it is clear that dysregulation of this relationship may cause chronic inflammation and/or metabolic disorders via bacterial stimulation of the host immune system (Turnbaugh et al., 2006; Wen et al., 2008; Garrett et al., 2010; Vijay-Kumar et al., 2010). Several animal model systems are introduced to dissect the molecular relationship between gut microbiota and gut inflammation (Koropatnick et al., 2004; Bates et al., 2007; Cani et al., 2008; Mazmanian et al., 2008; Ryu et al., 2008; Fraune et al., 2009; Kanther and Rawls, 2010). Although striking advances were made in recent years by taking advantage of technical innovations such as pyro-sequencing and omics technologies, the exact molecular mechanism of gut-microbiota interactions is only partly understood. This is probably due to the complexity of the host immune signaling pathways and also that of commensal community. *Drosophila*, a classical model for developmental biology and innate immunity, is now being introduced in the field of gut-microbiota interactions (Corby-Harris et al., 2007; Cox and Gilmore, 2007; Dietzl et al., 2007; Ren et al., 2007; Drysdale, 2008; Ryu et al., 2008; Apidianakis and Rahme, 2011; Chandler et al., 2011; Shin et al., 2011; Storelli et al., 2011; Wong et al., 2011; Broderick and Lemaitre, 2012; Charroux and Royet, 2012). Its elegant genetic tool box, simple commensal community, well-established knowledge on innate immune system, and easy to generate gnotobiotic animals make it possible to provide a novel insight on the dynamic dialog between bacterial and host cells. Genetic evidence demonstrated that reactive oxygen species (ROS), produced by dual oxidase (DUOX), a member of the intestinal nicotinamide adenine dinucleotide phosphate (NADPH) oxidase, are involved in diverse aspects of gut-microbe interactions, such as microbial clearance, intestinal epithelial cell renewal (ECR), redox-dependent modulation of signaling pathways, cross-linking of biomolecules, and discrimination between symbionts and pathogens. In the current review, recent advances on the regulation of DUOX in *Drosophila* gut as well as its role on the gut cell homeostasis and gut inflammation are discussed.

## Gut-interacting bacteria in *Drosophila*

Due to its open anatomical structure, gut epithelia are in constant contact with diverse ranges of microbial cells. These include resident “autochthonous” bacteria and also transiently passing “allochthonous” bacteria derived from the environment (Dillon and Dillon, 2004; Ley et al., 2008). In *Drosophila*, it is important to note that it is still unclear whether these resident autochthonous bacteria reside inside gut (i.e., stable colonization for a long time period) or transiently colonize gut (i.e., colonization for a short time period, but still longer persistence time when compared to that of transiently passing bacteria). Autochthonous symbionts (e.g., *Commensalibacter intestini*, *Acetobacter pomorum*, and *Lactobacillus plantarum*) constitute an important portion of resident bacteria that are believed to be beneficial to the host physiology (Ryu et al., 2008; Shin et al., 2011; Storelli et al., 2011). For example, *A. pomorum* and *L. plantarum* are known to enhance host development by stimulating important host signaling pathways such as insulin signaling and Tor signaling (Shin et al., 2011; Storelli et al., 2011). However, it is important to note that not all resident bacteria are symbiotic. For instance, *Gluconobacter morbifer* is considered a pathobiont, i.e., the resident bacterial species that is normally benign within a host, but can be conditionally pathogenic when commensal community is deregulated (Ryu et al., 2008). It has been shown that the pathobiont *G. morbifer* becomes pathogenic when the number of this bacterium exceeds a certain threshold following deregulation of gut immunity. In addition to these resident bacteria, the gut is also in contact with several other non-resident allochthonous bacteria that are introduced by the environment. *Erwinia carotovora* is a naturally occurring *Drosophila*-associated bacterium derived from the environment (Buchon et al., 2009b). *E. carotovora* is considered as an opportunistic pathogen because this bacterium does not harm the normal host but it can turn pathogenic when the host immune system is impaired (Ha et al., 2005a, 2009a,b). Among the allochthonous bacteria, certain species such as *Pseudomonas entomophila* and *Serratia marcescens*, are life threatening and thus classified as entomopathogens that are able to kill the host upon gut infection (Vodovar et al., 2005; Nehme et al., 2007). Therefore, it is evident that the host must draw maximum benefits from symbionts while antagonizing potentially pathogenic effects from pathogens and pathobionts, thereby achieving gut-microbiota homeostasis.

## Gut immunity in *Drosophila*

Due to the fact that the intestine harbors large amounts of bacterial cells, one of the most important questions is to understand the interactions between the host immunity and bacteria. Genetic analyses in *Drosophila* demonstrated that the gut epithelia are able to mount two distinct immune pathways: the immune deficiency (IMD) pathway that controls antimicrobial peptide (AMP) production, and the DUOX pathway that controls microbicidal ROS production (Lemaitre and Hoffmann, 2007; Bae et al., 2010; Royet et al., 2011; Buchon et al., 2013; Lee and Brey, 2013). As a plethora of excellent reviews on the IMD pathway, a *Drosophila* homolog of the mammalian NF-κB pathway can be found in several journals (Lemaitre and Hoffmann, 2007; Ganesan et al., 2010; Royet et al., 2011), the details on this pathway will not be described here. Several studies utilizing the IMD pathway mutant flies generated four interesting observations. First, the IMD pathway mutant flies are fairly resistant to gut infection, indicating that the IMD pathway is dispensable for the host resistance against gut infection in most cases (Ha et al., 2005a,b, 2009a,b). Second, chronic activation of the IMD pathway provokes modification of the gut commensal community, leading to the overgrowth of the opportunistic pathobionts (Ryu et al., 2008). Third, the IMD pathway mutant flies harbor higher amounts of gut microbiota (Buchon et al., 2009a). The second and third points indicate that the IMD pathway regulates the commensal community structure in a quantitative and qualitative manner. Finally, some bacteria that can subvert DUOX-dependent ROS are regulated by IMD-dependent AMPs, indicating that the IMD pathway likely plays a complementary role to the DUOX system, at least under certain circumstances (Ryu et al., 2010). In contrast to the IMD pathway mutant animals, animals with a reduced DUOX activity are highly susceptible to gut infection, indicating that DUOX-dependent ROS generation plays a major role in the control of gut-associated bacteria (Ha et al., 2005a; Bae et al., 2010). The DUOX system, particularly the diverse roles of DUOX in gut physiology, will be explored in further details.

## DUOX, a member of the NADPH oxidase family

**Figure 1:**
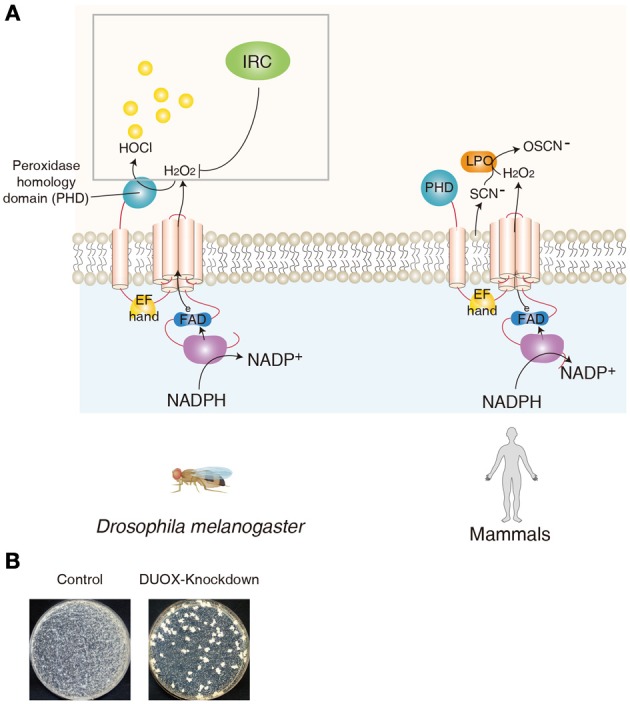
**DUOX as a mucosal antimicrobial system in *Drosophila* and human**. **(A)** Similar domains of DUOX enzymes between *Drosophila* and human are shown. In *Drosophila*, peroxidase homology domain of DUOX converts H_2_O_2_ into HOCl in the presence of chloride. DUOX-dependent H_2_O_2_ molecules are eliminated by immune-regulated catalase (IRC) activity. In human, DUOX-dependent H_2_O_2_ is used for the oxidative conversion of SCN^−^ to OSCN^−^ by the enzymatic action of lactoperoxidase in the mucosal fluids. **(B)** Modification of gut commensal community members in flies carrying reduced DUOX activity. Midgut of control flies and that of DUOX-knockdown flies are dissected and the homogenates of midguts are spread on Mannitol agar plate. Representative images are shown.

The role of ROS in the innate immune system was best illustrated by an oxidative burst in phagocytes (Babior, 2004). In this system, gp91^Phox^, a NADPH oxidase (now called NOX2), is responsible for the production of the superoxide anion (Segal, 2005). An analysis of the human genome sequence revealed several homologs of gp91^Phox^, now referred to as the NOX and DUOX family enzymes (Lambeth, 2004; Leto and Geiszt, 2006; Sumimoto, 2008). At present, five NOXs and two DUOXs have been identified in humans (Lambeth, 2004; Leto and Geiszt, 2006; Sumimoto, 2008), only one NOX and one DUOX homolog were observed in *Drosophila* (Donko et al., 2005; Ha et al., 2005a; Bae et al., 2010). These enzymes are found to be expressed in various non-phagocytic cells, including mucosal epithelial cells, suggesting novel physiological roles of ROS in diverse ranges of cells and tissues other than the phagocytes (Geiszt et al., 2003; El Hassani et al., 2005; Ha et al., 2005a; Allaoui et al., 2009; Fischer, 2009). Synthesis of the thyroid hormone in the thyroid gland is catalyzed by thyroperoxidase that requires the presence of H_2_O_2_, which is generated via the oxidation of NADPH by an NADPH oxidase in the thyroid (Dupuy et al., 1999; De Deken et al., 2000). DUOX was originally identified as a thyroid NADPH oxidase; however, it was later found to be expressed in the mucosal epithelia of the respiratory and gastrointestinal tracts (Geiszt et al., 2003; El Hassani et al., 2005). The DUOX gene is highly conserved amongst various organisms, from *Caenorhabditis elegans* to mammals (Edens et al., 2001; Ha et al., 2005a; Kawahara and Lambeth, 2007; Flores et al., 2010). The DUOX gene in the *Drosophila* genome is situated in the cytogenetic location 23B2-23B3, on the left arm of chromosome 2. The general structural organization of DUOX was well-conserved in all the studied organisms, and is presented in the Figure 1. The enzyme includes an extracellular peroxidase homology domain, a trans-membrane domain, a calcium-modulated EF hand domain, and a NADPH oxidase domain. Although the role of DUOX in the midgut has been most intensively studied, *DUOX* expression level in the midgut is found to be modest. High *DUOX* expression is observed in different organs in larvae (e.g., trachea, hindgut, and central nervous system) and adult (e.g., ovary, spermatheca, crop, and head) (see high-throughput expression data, such as FlyAtlas Anatomy Microarray analysis, in Flybase), suggesting distinct biological roles of DUOX in different organs.

## The role of DUOX in the oxidant-dependent antimicrobial response in epithelia

Following the identification of DUOX1/2 expression in the mammalian mucosal epithelia, several lines of evidence demonstrated that DUOX is a source of non-phagocytic ROS in the epithelial cells of the respiratory and gastrointestinal tracts (Geiszt et al., 2003; El Hassani et al., 2005). Because these cells function as a barrier that is in contact with microorganisms, it is believed that DUOX-dependent ROS may act as a microbicide, similar to phagocytic ROS. In this system, DUOX produces extracellular H_2_O_2_ that is used for the oxidative conversion of SCN^−^ to hypothiocyanate (OSCN^−^) by the enzymatic action of lactoperoxidase in the mucosal fluids (Leto and Geiszt, 2006; van der Vliet, 2008; Fischer, 2009) (Figure 1). Because hypothiocyanate can kill the bacteria, this DUOX-lactoperoxidase system is believed to provide a robust antimicrobial defense network in mammalian epithelial cells (Forteza et al., 2005; Boots et al., 2009; Gattas et al., 2009). However, because all of these observations in the mammalian system were made in *in vitro* cultured primary cells/tissues or cell lines, the precise *in vivo* role of DUOX in the host antimicrobial defense in an organism remains to be elucidated in mammals. The most direct evidence on the *in vivo* role of DUOX was first provided in a *Drosophila* gut infection model system (Ha et al., 2005a). As mentioned earlier, in contrast to the essential role of AMP-based immunity when microorganisms enter the body (i.e., systemic infection), AMP-based immunity plays only a minor role when microorganisms are introduced in the gut by oral ingestion (i.e., gut infection). For example, AMP-deficient mutant animals are apparently healthy following a gut infection, suggesting the existence of other immune systems that can regulate the bacteria in the gut epithelia (Ha et al., 2005a,b). It was demonstrated that DUOX-knockdown (KD) flies are highly susceptible to gut infections by various microorganisms. Tissue-specific KD experiments showed that the DUOX activity in the gut epithelia is responsible for host resistance to gut infection (Ha et al., 2009b). Additional biochemical studies showed that DUOX is the source of infection-induced ROS in *Drosophila* gut (Buchon et al., 2009a; Ha et al., 2009a,b). Later, the importance of DUOX in gut immunity was also demonstrated in the *C. elegans* and zebrafish model systems (Flores et al., 2010; Hoeven et al., 2011). Although DUOX-mutant mice are available, they exhibit pleiotropic phenotypes such as dwarfism, which makes it difficult to unambiguously conclude the role of DUOX in this animal model (Johnson et al., 2007). Further analysis using conditional knockout animal models will be necessary to validate the *in vivo* role of DUOX in mucosal immunity.

How does *Drosophila* DUOX antagonize bacterial growth *in vivo*? It has been suggested that the NADPH oxidase domain of DUOX produces H_2_O_2_ in the gut lumen, and a peroxidase homology domain, the second domain of DUOX, converts H_2_O_2_ into HOCl in the presence of chloride (Ha et al., 2005a) (Figure 1). In support of this notion, the recombinant peroxidase homology domain can kill the bacteria only in the presence of both H_2_O_2_ and chloride (Ha et al., 2005a).

In the absence of gut infection, the metazoan gut harbors significant amounts of bacterial cells under conventional conditions (Ley et al., 2008; Lee and Lee, 2013). This commensal community structure (both in terms of bacterial diversity and density) is known to be actively shaped by the host immunity (Artis, 2008; Pedron and Sansonetti, 2008; Ryu et al., 2008; Round and Mazmanian, 2009; Cerf-Bensussan and Gaboriau-Routhiau, 2010; Littman and Pamer, 2011; Maslowski and Mackay, 2011; Hooper et al., 2012; Lee and Lee, 2013). It has been shown that a regulated level of IMD pathway potential is essential for a normal commensal community structure (Ryu et al., 2008). As the DUOX system is the primary host immune system that provides a robust antimicrobial response in the microbe-laden epithelia in metazoans, it is expected that the loss-of-DUOX activity would result in dysregulation of the commensal community (Ha et al., 2009a). On examination of the gut microbiota of DUOX-KD flies cultured in a growth plate, it is consistently observed that the gut commensal community of DUOX-KD flies is highly modified, as evidenced by the presence of higher bacterial cell number, different shapes of bacterial colonies, and the presence of fungi (Ha et al., 2009a) (Figure 1). This indicates that the absence of a major defense system leads to a severe dysregulation of the gut-associated microbiota. Given that DUOX-KD flies under conventional (CV) conditions had a short life span that could be completely rescued under germ-free (GF) condition (Ha et al., 2009a), and that the monoassociation of DUXO-KD flies with each of the resident symbiotic bacteria did not affect their survival rate, the dysregulated commensal community may be the direct cause of mortality. However, opportunistic pathogens and/or pathobionts responsible for the lethality of conventional DUOX-KD flies remain to be elucidated.

Unlike AMPs specific to prokaryotic cells, microbicidal ROS are also cytotoxic to eukaryotic host cells. Therefore, ROS production must be tightly regulated to avoid excess oxidative stress. It was found that flies lacking secretory immune-regulated catalase (IRC) showed high lethality against gut infection due to oxidative stress (Ha et al., 2005b) (Figure 1). As IRC possesses a H_2_O_2_-scavenging activity, this observation indicates that infection-induced ROS are dynamically removed by IRC. Therefore, it is likely that DUOX-dependent ROS generation and IRC-dependent ROS removal modulate redox-dependent innate immunity to antagonize pathogen growth, while protecting host cells from an excess immune response (Ha et al., 2005a,b).

## Microbial ligands for DUOX activation

**Figure 2:**
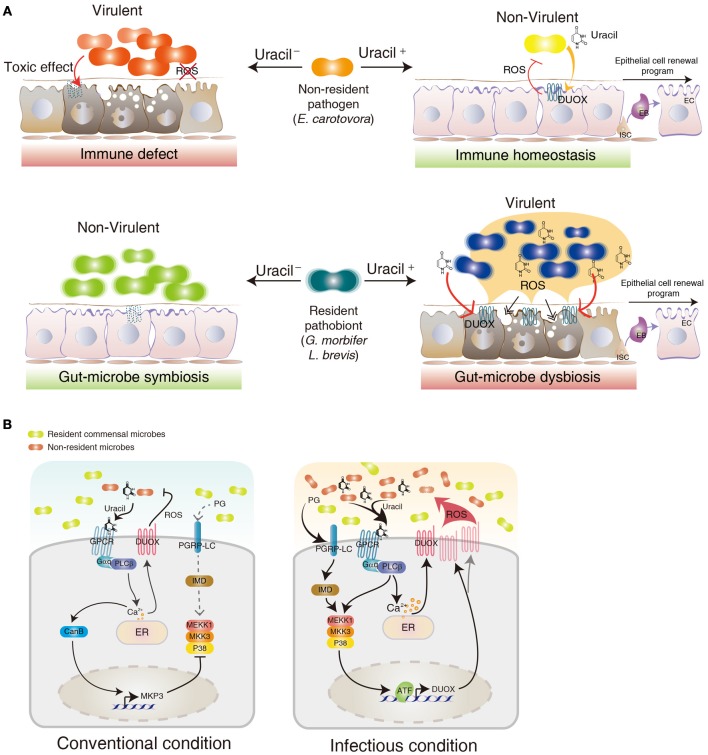
**Role of DUOX in gut-microbe interactions. (A)** Different gut physiologies depending different uracil-releasing states (Uracil^−^ and Uracil^+^ for uracil non-releasing and releasing state, respectively) and different gut-colonizing ability (resident vs. non-resident) of each bacterium in a *Drosophila* gut environment. **(B)** DUOX regulatory mechanism in conventional and infectious conditions. See text for more details.

The identification of the DUOX system in the gut epithelia raises an important question of how a host senses different bacteria to induce DUOX activation. In *Drosophila*, meso-diaminopimelic acid-type peptidoglycan (PG) primarily released from Gram-negative bacteria acts as an agonist for the IMD activation in the gut (Leulier et al., 2003; Royet et al., 2011). However, PG was unable to induce a DUOX-dependent ROS generation, indicating that ligands other than PG (non-PG ligands) are derived from the bacteria to induce DUOX activation (Ha et al., 2009a,b; Bae et al., 2010). Because most microorganisms, including yeast and Gram-positive bacteria, can also activate the DUOX system, these non-PG ligands are believed to commonly exist in diverse microorganisms. In contrast to the robust DUOX activation following gut epithelial contact with allochthonous bacteria, most symbiotic autochthonous bacteria do not cause DUOX activation (Lee et al., 2013). This observation suggests that non-PG ligands may acts as pathogen-specific ligands that may be absent and/or reduced in symbionts, allowing a distinction between allochthonous and autochthonous bacteria. It has recently been found that this non-PG ligand is indeed secreted from allochthonous bacteria but not from the autochthonous bacteria (Lee et al., 2013). Chemical analyses of this non-PG ligand have revealed that it is a uracil nucleobase. Synthetic uracil is found to be very capable of stimulating DUOX activation (range approximately 100 pM–100 nM) whereas other nucleobases are inefficient ligands under similar concentrations. Furthermore, uracil is unable to activate the IMD pathway, indicating that uracil-based immunity is distinct to PG-based immunity (Lee et al., 2013). This uracil-based immune system is unique because PG-based immune systems fail to distinguish between pathogens and symbionts because both bacteria have a similar capacity to induced the PG-dependent IMD pathway (Lee et al., 2013). All of these observations suggest that the gut epithelia selectively mount DUOX activation by sensing pathogen-derived uracil. Mutant pathogens with reduced uracil secretion (e.g., uracil auxotrophic *E. carotovora* strain) could avoid DUOX activation with this being lethal to the host, whereas the wild type *E. carotovora* strain would not harm the normal host (Lee et al., 2013) (Figure 2). These observations demonstrate that the recognition of pathogen-derived uracil is essential for the control of opportunistic pathogens such as *E. carotovora* and host survival. These observations also raise the interesting possibility that a reduction of uracil secretion may be employed as a virulence mechanism for the pathogen to avoid host immunity (Figure 2). It would be interesting to see whether host-killing *Drosophila* pathogens use this strategy to avoid the host DUOX system.

As uracil can be found in any living cells including symbiotic or pathogenic bacteria, it is presently unclear why symbiotic bacteria do not secret uracil whereas pathogens do so. The mechanism of uracil secretion from the bacteria is presently unknown. The secretion of uracil in the case of *E. coli* is only observed when growth conditions are unfavorable, e.g., in response to entry into the stationary phase or to a perturbation of balanced growth conditions (Rinas et al., 1995). This observation indicates that uracil release is controlled by the bacterial cells depending on the environmental conditions. It is unclear why bacteria release uracil under unfavorable condition. One interesting possibility is that it may act as a bacterial survival signal to overcome the stringent conditions. For example, *Pseudomonas aeruginosa* can respond to exogenous uracil by reprogramming the bacterial gene expressions involved in virulence, quorum sensing, and biofilm formation (Ueda et al., 2009). Therefore, one can speculate that uracil release is a normal bacterial response to resist stressful conditions; this is beneficial for the survival of bacterial cells. In this context, it is possible that gut environments are stressful conditions for most environment-derived opportunistic pathogens which initiate uracil release *in situ* to promote their survival. However, this survival strategy is potentially dangerous to the host cells. Therefore, host may have evolved to sense the bacterial status from uracil presence, subsequently antagonizing pathogens before they mount their survival strategy. Another interesting point is that, as uracil can be also found in any eukaryotic cells, it may act as a danger signal released from damaged host cells. In this case, it is possible that host could mount innate immunity by sensing uracil released from host cells damaged by pathogens (e.g., by intracellular pathogens). Further detailed investigations of all these interesting possibilities will be needed to better understand the complex interactions between host immunity and different gut-associated autochthonous/allochthonous bacteria.

Monoassociation of GF animals with each type of commensal bacteria revealed that most symbiotic autochthonous bacteria do not elicit a DUOX activation probably due to the absence of uracil release (Lee et al., 2013; Valanne and Ramet, 2013). This observation indicates that symbiotic autochthonous bacteria may have evolved to adapt to the gut environment by avoiding DUOX activation possibly by modifying the pathway of uracil secretion. However, some resident bacteria, such as *G. morbifer* and *L. brevis*, do induce a chronic DUOX activation, suggesting that these gut-dwelling pathobionts may chronically release the uracil that is responsible for the chronic DUOX activation (Lee et al., 2013) (Figure 2). Chronic DUOX activation results in gut cell apoptosis and early host death, which is reminiscent of the phenotypes found in chronic inflammatory diseases. The reduction of uracil release by generating URA^−^ mutant pathobionts is sufficient to prevent all the disease phenotypes, with a resulting bacterial phenotypic shift from pathobionts to symbionts (Lee et al., 2013) (Figure 2). These observations demonstrate that uracil release from gut-dwelling bacteria can act as a virulence factor of the opportunistic pathobionts. It is presently unknown why pathobionts are generally benign within a normal commensal community but become pathogenic under certain conditions. If uracil excretion can be controlled by the bacteria in a context-dependent manner, one intriguing possibility is that pathobionts can become pathogenic when they initiate their uracil secretion pathway under certain dysregulated gut environments (Figure 2). Future studies on the mechanism of the uracil secretion pathway and its differential regulation between the symbiont and pathobionts will be needed to better understand the physiological characteristics of pathobionts and symbionts.

Interestingly, uracil can also stimulate DUOX activation in *C. elegans* as well as in human bronchial and intestinal epithelial cells (Lee et al., 2013). It would be interesting to investigate whether the uracil-mediated DUOX activation mechanism is involved in the etiology and pathogenesis of mammalian epithelial inflammatory diseases that arise from abnormal mucosa-microbe interactions.

## The DUOX regulatory mechanism

Gut epithelial cells are in continuous contact with basal amounts of bacterial ligands such as PG and uracil (Lee and Lee, 2013). As chronic and/or overactivation of the DUOX system may lead to a deleterious effect on host cells, DUOX activation must be tightly regulated to avoid oxidative damages while preserving intact microbicidal activity (Ha et al., 2009b; Lee and Lee, 2013). At present, genetic analyses have revealed that two signaling pathways are controlling DUOX-dependent ROS generation (Ha et al., 2009b). The DUOX-activity pathway composed of PLCβ-calcium signaling is responsible for the induction of DUOX enzymatic activity whereas the DUOX-expression pathway composed of the MEKK1-MKK3-p38 MAPK-ATF2 transcription factor is responsible for the induction of DUOX gene expression (Ha et al., 2009b) (Figure 2).

It is known that these two pathways are differentially activated depending on the local microbial burdens. By comparing the GF animals (devoid of any bacterial cells) and CV animals (having normal symbiotic microflora as well as some environment-derived microorganisms) it was found that CV animals consistently showed higher basal ROS levels than those found in GF animals or GF animals monoassociated with symbiotic commensal bacteria (Lee et al., 2013). This observation indicates that gut-associated microflora other than symbionts found in the CV environment stimulates basal levels of DUOX activity. Basal levels of DUOX are known to be required for the routine control of gut-introduced microorganisms such as dietary yeast, *Saccharomyces cerevisiae* (Ha et al., 2009b). In this condition, basal PLCβ activity induces low calcium mobilization to maintain the basal DUOX activation because the DUOX enzyme is dependent on calcium concentration (Figure 2). When gut epithelia are further subjected to gut infection, the PLCβ-calcium signaling becomes maximally activated to induce full DUOX activity (Ha et al., 2009b) (Figure 2). It is important to note that this PLCβ-calcium signaling is activated by uracil but not by PG, indicating that the IMD pathway and the DUOX pathway are distinct (Lee et al., 2013). As a variety of microbial cells can induce DUOX activation, it is likely that uracil is released from many microbial cells in the gut. Under infectious conditions, the DUOX-expression pathway becomes activated by two different bacterial ligands, uracil, and PG (Ha et al., 2009b; Lee et al., 2013) (Figure 2). Uracil activates MEKK1-MKK3-p38 in a PLCβ-dependent manner possibly by PKC activation, whereas PG activates MEKK1-MKK3-p38 in a PGRP-LC and IMD-dependent manner (Figure 2). It should be noted that MEKK1 mutant animals having an intact DUOX-activity pathway but impaired DUOX-expression pathway survive normally under CV conditions (Ha et al., 2009b). They are, however, highly susceptible to gut infections. These observations indicate that the DUOX-activity pathway alone is required and sufficient for the control of routine microbial burdens whereas both DUOX-activity and the DUOX-expression pathway are required for the control of high microbial burdens.

It is important to note that the basal DUOX-activity pathway is required for the inhibition of the DUOX-expression pathway under CV conditions (Ha et al., 2009a,b; Bae et al., 2010) (Figure 2). For example, PLCβ mutant flies showed constitutive p38 MAPK activation and DUOX gene overexpression under CV conditions but not GF conditions (Ha et al., 2009a). It has been shown that basal PLCβ-calcium signaling induces calcium-dependent calcineurin B and MAPK phosphatase 3 (MKP3) gene expression (Ha et al., 2009b) (Figure 2). MKP3 negatively regulates p38 phosphorylation. As the calcineurin inhibitor FK506 abolished MKP3 gene expression, Calcineurin B acts as an upstream component of MKP3 (Ha et al., 2009b). MKP3-KD flies having a high DUOX-expression pathway activation exhibited a short life span under CV conditions due to oxidative stress, indicating that the negative regulation of the DUOX-expression pathway by the DUOX-activity pathway is required to avoid excess oxidative stress under routine gut-microbe interactions (Ha et al., 2009b; Bae et al., 2010).

## DUOX in gut integrity

**Figure 3:**
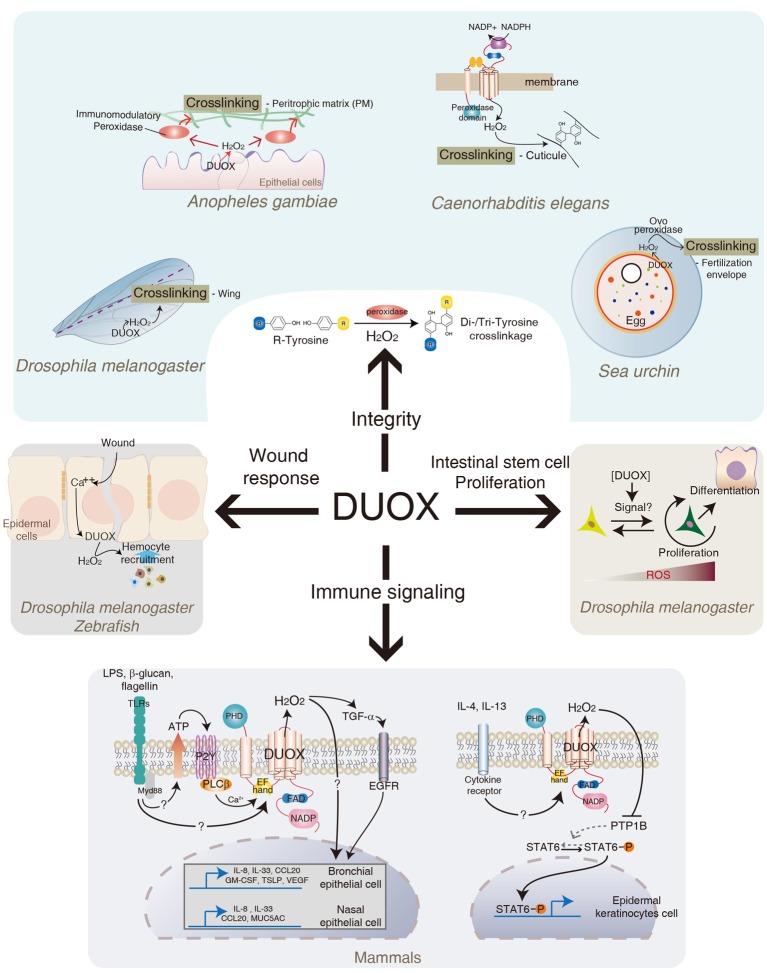
**Role of DUOX in diverse biological activities**. In addition to its original function in redox-dependent antimicrobial defense in mucosa described in Figures 1, 2. DUOX system is also involved in cross-linking of biomolecules, intestinal epithelial cell renewal, redox-dependent modulation of signaling pathways, and wound healing in different metazoans. See text for more details.

In addition to its direct microbicidal actions, other interesting aspects of DUOX are also documented (Figure 3). In *Anopheles gambiae*, DUOX is known to be involved in gut permeability by forming a dityrosine network of the peritrophic membrane, a non-cellular semi-permeable layer of chitin polymers covering the midgut epithelia (Kumar et al., 2010). In this system, DUOX-dependent H_2_O_2_ acts as a substrate of secreted heme peroxidase that catalyzes protein cross-linking in the mucin layer. In an *Anopheles* with reduced DUOX expression, gut permeability increases due to the reduction of dityrosine cross-linking of the peritrophic membranes (Kumar et al., 2010). It was shown that DUOX activity mediates cross-linking between macromolecules, e.g., between collagen and other proteins, via di- and tri-tyrosine linkage, for the formation of the cuticular extracellular matrix in *Caenorhabditis elegans* (Edens et al., 2001). In the sea urchin eggs, DUOX-dependent H_2_O_2_ is shown to be essential for the oxidative cross-linking of the fertilization envelop (Wong et al., 2004). Similarly, *Drosophila* DUOX was found to be involved in the stabilization of the adult wing, possibly by tyrosine cross-linking (Anh et al., 2011). Therefore, bacterial-induced DUOX activity may regulate the formation of a physical barrier such as the peritrophic membrane that provides a buffered zone between commensal bacteria and enterocytes. In this regard, it is interesting to note that DUOX-KD flies under CV condition showed spontaneous IMD pathway activation when the flies became old (Lee and Lee, Unpublished observation), which was abolished in GF DUOX-KD flies. These results suggest that increased peritrophic membrane permeability and/or increased bacterial burden observed in DUOX-KD flies are responsible for spontaneous IMD pathway activation. Further studies will be needed to elucidate the exact cause of spontaneous IMD pathway activation in aged DUOX-KD flies. In mammals, DUOX is known to be involved in the expression of MUC5AC mucin, one of the major components of airway mucus, in the airway epithelia in response to different stimuli (Shao and Nadel, 2005). In this case, DUOX-dependent H_2_O_2_ acts as a second messenger to modulate signaling pathways, leading to MUC5AC expression, although the exact mechanisms remain to be elucidated. In the *Drosophila* genome, 17 mucins and 19 mucin-related proteins are identified (Syed et al., 2008). It would be interesting to see whether DUOX activity also mediates the expression of these mucins in the midgut epithelia.

## DUOX in intestinal stem cell activation

The process of gut infection introduces a high density of bacterial cells into the gut lumen, which inevitably damages the epithelial cells lining the intestinal tract. These damaged cells need to be replaced by newly emerged cells to maintain gut cell homeostasis. It was recently shown that bacterial infection induces an ECR program that is responsible for replenishing the damaged cells (Amcheslavsky et al., 2009; Buchon et al., 2009a,b; Chatterjee and Ip, 2009; Cronin et al., 2009; Jiang et al., 2009). This ECR program includes intestinal stem cell (ISC) proliferation and differentiation. Although the ECR program controls the normal turn-over rate of gut epithelial cells, the infection process accelerates the ECR program due to the massive gut cell loss (Buchon et al., 2009a,b, 2010; Chatterjee and Ip, 2009; Jiang et al., 2009). Upon gut infection, each ISC produces one daughter cell that retains the fate of its parent cell, and one postmitotic enteroblast that in turn differentiates into either an enterocyte or an enteroendocrine cell (Micchelli and Perrimon, 2006; Ohlstein and Spradling, 2006, 2007). Several signaling pathways such as growth factor signaling and JAK-STAT signaling pathways are known to be involved in the ECR program (Buchon et al., 2009b, 2010; Cronin et al., 2009; Jiang and Edgar, 2009; Jiang et al., 2009; Xu et al., 2011; Zhou et al., 2013). Interestingly, flies with reduced DUOX activity fail to mount a normal ECR program following gut infection, as evidence by reduced ISC proliferation and differentiation (Buchon et al., 2009a). Based on this result, it has been proposed that DUOX-dependent ROS molecule is one of major inducers to initiate the ECR program. Given that ingestion of tissue damaging agents such as sodium dodecyl sulfate or paraquat could initiate ECR, it is speculated that the increase in the ECR program is not a direct effect of ROS but rather an effect of the ROS-induced host cell damage (Buchon et al., 2009a). Alternatively, DUOX-dependent ROS molecule may act as a direct signaling molecule to initiate ECR program. It is routinely observed that the DUOX-KD flies exhibited a higher gut cell apoptosis index in a CV condition when compared to that observed in control flies; i.e., more than 2% in 13-day-old DUOX-KD flies vs. less than 0.2% in control flies of the same age (unpublished observation). Despite the high gut cell apoptosis index, these DUOX-KD flies demonstrated a reduced rate of ECR program, raising an alternative possibility in that a certain level of ROS acts as a critical signal to initiate the ECR program. In agreement with this notion, recent evidences showed that ISCs in *Drosophila* are under redox-control and that reduced ROS level favors stemness whereas elevated ROS level initiates the differentiation program (Biteau et al., 2008; Buchon et al., 2009a; Lee, 2009; Owusu-Ansah and Banerjee, 2009; Hochmuth et al., 2011; Jasper and Bohmann, 2013) (Figure 2). It has been proposed that different ROS levels modulate the specificity and intensity of the signal response as well as the adhesive properties of stem cells within a niche. Interestingly, *L. plantarum*, but not other bacterial species, was recently shown to induce NOX-dependent ROS to modulate ECR program in *Drosophila* (Jones et al., 2013). In the study of interactions between gut and a specific bacterium, it is important to note that bacterial micro-diversity within the same species even with 100% identical 16S rRNA was reported in many bacteria (Jaspers and Overmann, 2004). Distinct physiology, such as phenotypic and genomic diversity, among different strains of the same species, *L. plantarum*, was also reported (Siezen et al., 2010). For example, a recent report showed that a *L. plantarum* IBDML1 strain is unable to promote *Drosophila* larval growth whereas a *L. plantarum* strain WJL strain can promote larval development under the same experimental conditions (Storelli et al., 2011), indicating that the physiological characteristics of microorganisms should be studied in a strain level, but not in a species level. Therefore, it is possible that each bacterial strain may differentially influence ECR program by activating distinct enzymes (i.e., NOX or DUOX) with different mode of enzyme activation in terms of intensity and duration. This important issue can be clarified by clearly establishing the ROS-inducing mode of each bacterial strain and the molecular mechanisms by which ROS modulate intracellular signaling pathways involved in ISC proliferation and differentiation. The ingestion of uracil is sufficient to induce all aspects of the ECR program such as ISC proliferation and differentiation as well as JAK-STAT activation (Lee et al., 2013). Thus, the uracil-induced ECR program will provide a unique opportunity to dissect the molecular mechanism by which DUOX modulates ISC regulation.

## DUOX in signal transduction

Although H_2_O_2_ is a well-known cytotoxic molecule that can damage the host, it became evident that the physiological concentration of H_2_O_2_ is essential for the relay of many important intracellular signaling pathways (Sauer and Wartenberg, 2005; Rhee, 2006; Stone and Yang, 2006). In this regard, it is interesting to note that DUOX is found to be activated following ligand-dependent stimulation of TLRs in mammals (Figure 3). For example, interactions between the microbial components and TLRs, such as flagellin/TLR5, LPS/TLR4, and β-1,3-glucan/TLR2, are shown to induce DUOX activation in human airway epithelial cells (Koff et al., 2008; Joo et al., 2012; Ryu et al., 2013) (Figure 3). However, the mechanism by which TLR stimulation leads to DUOX activation is less clear. Co-immunoprecipitation experiments showed that DUOX is physically associated, directly or indirectly, with at least some members of the TLR family, such as TLR2 and TLR5 (Joo et al., 2012; Ryu et al., 2013). One possibility is that this TLR stimulation following ligand binding may induce structural changes of TLR, which somehow contributes to the DUOX activation state. Alternatively, TLR stimulation induces DUOX activation by intracellular calcium mobilization. For example, upon TLR stimulation, cells release ATP that induces PLCβ-dependent calcium mobilization via purinergic receptor activation (Boots et al., 2009) (Figure 3). As calcium mobilization can directly modulate the DUOX enzyme activity via its EF-hand domains, it can be speculated that bacterial ligands capable of inducing calcium, directly or indirectly, could induce calcium-dependent DUOX activation and H_2_O_2_ production. Importantly, the absence of DUOX-dependent H_2_O_2_ production abolished the expression of TLR-downstream target genes in epithelial cells, such as IL8 and Mucin 5AC, and CCL20 chemokines, highlighting the importance of DUOX-dependent H_2_O_2_ in TLRs signaling pathways (Koff et al., 2008; Joo et al., 2012; Ryu et al., 2013). It is presently unclear how DUOX-dependent H_2_O_2_ contributes to the expression of inflammatory genes in epithelial cells. One possible mechanism is that DUOX-dependent H_2_O_2_ somehow converts the latent form of TNF-α converting enzyme (TACE) to its active form, which in turn cleaves the proform of TGF-α to its active form (Koff et al., 2008). The active form of TGF-α in turn induces EGFR signaling activation to induce inflammatory gene expression such as IL8. However, other H_2_O_2_-dependent and ligand-independent EGFR activations are also described (Boots et al., 2009). In this system, DUOX-dependent H_2_O_2_ activates Src kinase, which in turn activates EGFR in a ligand-independent manner. In *Drosophila* and zebrafish, DUOX-dependent H_2_O_2_ production in response to tissue injury is shown to be critical to attract hemocyte recruitment and wound repair gene expression (Niethammer et al., 2009; Moreira et al., 2010) (Figure 3). Epithelial injury in *Drosophila* embryo induces DUOX-dependent ROS generation that is in turn required for the induction of ERK-dependent wound repair genes such as *dopa decarboxylase* and *tyrosine hydrolase* (Juarez et al., 2011; Razzell et al., 2013). How does H_2_O_2_ modulate such diverse signaling pathways? It is well-known that H_2_O_2_ can modify protein structure and function by the oxidation of some amino acid residues such as cysteine (Stadtman and Levine, 2003). Several redox-regulated signaling molecules have been documented (Veal et al., 2007). These include transcription factors (e.g., c-Jun/c-Fos, Nrf-2/Keap-1), several kinases (JNK, MEKK1, I-κB kinase, Src tyrosine kinase), and phosphatase (e.g., PTEN and PTP). Indeed, it has been shown that the Th2 cytokines, IL4 and IL13, induce DUOX-dependent ROS generation in normal human epidermal keratinocytes, and that DUOX-dependent ROS induces oxidative inactivation of the catalytic cysteine 215 of the protein tyrosine phosphatase 1B (Hirakawa et al., 2011). Inactivation of protein tyrosine phosphatase 1B acts as a positive feedback loop that prolongs the duration of IL4- and IL13-induced STAT6 phosphorylation (Figure 3). Given that DUOX activation acts genetic upstream of JAK-STAT activation during ISC differentiation in *Drosophila*, it would be interesting to examine whether a similar mechanism operates in the ECR program in *Drosophila* gut epithelia. In sum, all the relevant evidences suggest that the ligand-dependent generation of physiological concentration of DUOX-dependent H_2_O_2_ likely plays a critical role in the initiation and amplification of diverse signaling pathways, including inflammatory and wound repair signaling. The identification of target redox-regulated signaling molecules controlled by DUOX-dependent H_2_O_2_ will clearly elucidate the exact molecular mechanism of DUOX-mediated signaling pathways.

## Conclusion

Signal-dependent ROS productions are now considered to play a pivotal role in a diverse range of host physiology. Genetic studies using the *Drosophila* model system unambiguously demonstrated the *in vivo* role of mucosal DUOX on bacterial control (Ha et al., 2005a). Strikingly, its unique mode of activation by bacteria-derived uracil makes it possible to distinguish between bacteria that release uracil and bacteria that cannot (Lee et al., 2013). Considering that the uracil-releasing ability and gut-colonizing ability of each bacterium determines the total amount and duration of uracil released *in situ*, respectively, these two bacterial characteristics are the factors controlling the intensity of DUOX activity *in vivo*. Insufficient DUOX activation by allochthonous bacteria may result in an infectious condition, whereas long-term DUOX activation by autochthonous bacteria may lead to chronic inflammation (Lee et al., 2013). In this regard, it is important to investigate the bacterial mechanism of uracil release and its regulation in different bacteria. This information may provide a novel insight on the molecular mechanisms of gut-microbe symbiosis and gut-microbe pathogenesis. It is also exciting to observe diverse DUOX functions in the mucosal epithelia. In addition to its antimicrobial response, it becomes evident that DUOX plays a central role in gut permeability and modulation of signal transductions involved in immune gene expression, wound healing, and stem cell regulation. Biochemical analyses on the identification of redox-controlled signaling molecules will provide a clearer picture on the mechanism of DUOX-modulated signaling pathways. One issue however remains; the host receptors responsible for DUOX activation. Analysis on the DUOX-activating signaling pathway revealed that G-protein coupled receptors (GPCRs) are involved in the recognition of bacterial ligands or other stimuli to initiate DUOX activation (Ha et al., 2009a; Lee et al., 2013). Approximately 300 GPCRs have been identified in the *Drosophila* genome (Brody and Cravchik, 2000; Hewes and Taghert, 2001). Preliminary genetic screening revealed that multiple GPCRs seem to be involved in the DUOX activation during gut-microbe interactions. The identification and characterization of these GPCRs and their respective ligands will provide a better understanding of the mechanism of how gut epithelia sense environmental ligands for DUOX activation, and of how each GPCR contributes to DUOX-modulated gut physiology.

### Conflict of interest statement

The authors declare that the research was conducted in the absence of any commercial or financial relationships that could be construed as a potential conflict of interest.
